# Structural and Functional Stability of Strontium Aluminate-Based Luminescent Composites After 15 Years of Natural Weathering (Indoors and Outdoors)

**DOI:** 10.3390/polym18161949

**Published:** 2026-08-09

**Authors:** Mª Ángeles Rodríguez-González, Natalia Díaz-Rodríguez, Miguel Rubio-Carrizo, Fausto Rubio

**Affiliations:** 1 Environmental Resources Analysis Research Group, University of Extremadura, 06006 Badajoz, Spain; 2 Biochemistry and Molecular Biology and Genetics Department, University of Extremadura, 06006 Badajoz, Spain; nataliadr@unex.es; 3 Chemistry-Physics of Surfaces and Processes Department, Institute of Ceramics and Glass (CSIC), 28049 Madrid, Spain; miguel.rubio@icv.csic.es (M.R.-C.); frubio@icv.csic.es (F.R.)

**Keywords:** natural weathering, indoor, outdoor, luminescence, strontium aluminate, functional stability

## Abstract

SrAl_2_O_4_: Eu^2+^, Dy^3+^ polymeric composites are the reference photoluminescent materials in passive signaling due to their high emission intensity and long luminescent persistence. But their real long-term outdoor durability is still poorly understood. This study analyzes a strontium aluminate polymer composite exposed to real weathering for 15 years to evaluate its degradation threshold, exceeding the frameworks of accelerated tests. Using FTIR, Raman, FE-SEM, colorimetry and phosphorescence decay, the weathered material was compared with its reference material. Results show that the luminescent composite retains its structural properties, its optical functionality improving its mechanical properties (increasing the flexural strength between 23 and 64% and microhardness between 136 and 164%), while suffering only a small yellowing, suggesting a longer service life than expected. Finally, it was established that the loss in the optical performance is not due to irreversible degradation of the polymer or pigment but is caused by the accumulation of surface dust and the formation of an opaque outer layer. The application of mechanical surface polishing removes this polluting layer, restoring optical transmittance and effectively recovering almost the original luminosity of the photoluminescent system.

## 1. Introduction

Persistent luminescent materials have been studied for decades as inorganic systems capable of storing excitation energy and releasing it gradually after the excitation source is removed [1,2,3]. Their afterglow is generally understood in terms of carrier trapping and de-trapping at defect-related energy levels, although the detailed mechanism remains system-dependent and not fully resolved in all hosts [1,4,5]. Trap depth and trap-depth distribution are central parameters because they govern the energy required to release stored carriers and therefore strongly influence persistent emission [2,6].

Research in this field accelerated sharply after the discovery of the bright green long afterglow SrAl_2_O_4_:Eu^2+^,Dy^3+^ in the mid-1990s, which established this composition as a benchmark persistent phosphor [2,5,7]. SrAl_2_O_4_:Eu^2+^,Dy^3+^ combines high brightness and long persistence with broad practical relevance, and it remains the most extensively studied material in this class [8,9]. Its green emission is typically assigned to the 4f^6^ 5d^1^ → 4f^7^ transition of Eu^2+^, reported near 506–525 nm depending on synthesis route and microstructure [7,8,10]. Subsequent studies showed that Dy-related trap formation, defect engineering, and synthesis control strongly affect brightness, decay kinetics, and persistence time [7,9].

At the same time, mechanistic work has shown that persistent luminescence in strontium aluminate cannot be reduced to a single simple trap picture. Reviews now emphasize multiple models involving defect populations, oxygen vacancies, conduction-band processes, and trap manipulation strategies [3,4,6]. In SrAl_2_O_4_-based systems specifically, oxygen vacancies have been linked to enhanced afterglow, while recent work also shows that deep traps, although not directly responsible for room-temperature emission, can strongly alter the filling dynamics of the shallow traps that control visible afterglow [5,11]. This mechanistic complexity is important when comparing materials processed by different routes, since particle size, crystallinity, and phase purity can modify both emission and decay behavior [8,9,12].

In parallel, long time environmental durability of polymer matrices has long been recognized as a critical issue for outdoor photonic and structural composites. Polymeric materials degrade under combined exposure to ultraviolet radiation, temperature, and humidity, with deterioration in chemical structure and mechanical performance driven by photo-oxidative and thermo-oxidative processes [13] and air pollutants like NO_x_ and SO_x_ [14]. Accelerated aging studies further show that degradation kinetics depend strongly on irradiation wavelength, irradiance, and temperature, and that unrealistic UV spectra can trigger mechanisms not representative of terrestrial weathering [15].

This durability problem becomes more complex in luminescent composites, where matrix aging, interfacial damage, and luminophore stability can interact. Recent polymer–phosphor studies show that photoluminescent fillers can be used to sense UV-induced degradation in polyester composites, while other luminescent composite systems demonstrate that matrix selection and encapsulation strategy are decisive for resistance to moisture, chemicals, and long-term optical loss [16,17]. Hydrophobic or chemically resistant matrices can substantially improve stability in humid or aqueous environments by limiting water interaction and suppressing quenching or degradation pathways [18,19].

From this perspective, the long-term outdoor behavior of SrAl_2_O_4_:Eu^2+^,Dy^3+^-based polymer composites should be interpreted at the intersection of three established research lines: the defect-controlled physics of persistent luminescence, the historical development of strontium aluminate as a benchmark afterglow phosphor, and the environmentally driven aging of polymer matrices under realistic weathering conditions [1,9,13]. A 15-year natural exposure study is therefore valuable not only as an application-oriented durability test, but also as a rare long-timescale experiment linking phosphor stability, matrix degradation, and service-environment effects in a single material system [9,16].

The hypothesis of this work states that, after 15 years of environmental exposure, the composite will have undergone differential degradation, but coupled between its two constituent phases. On the one hand, the optical transmittance of the system will have been reduced due to photo-yellowing, generation of oxidative species, and microcracking of the polyester resin [20,21]. On the other hand, the diffusion of water and the hydrothermal action continued through the matrix over a decade and a half will have favored hydrolysis and degradation processes at the resin-particle interface, compromising the structural stability of strontium aluminate, whose sensitivity to moisture and need for encapsulation have been widely recognized [14,22].

In order to test this hypothesis and quantify the impact of natural aging, this work carries out a direct comparison between the material exposed during 15 years in service and its corresponding reference material in the initial state, following approaches similar to those applied in recent studies comparing aged and unaged specimens through mechanical tests and chemical and morphological characterization techniques [23,24,25]. The research is articulated from a multiscale and multidisciplinary approach, trying to correlate the macroscopic response of the material (mechanical resistance, fatigue or creep behavior and structural integrity) with the microstructural and chemical alterations identified by advanced characterization techniques, in line with works that integrate micro/macro analysis and modeling of damage in polymers and composites subjected to environmental aging [22,23,24].

First, the chemical degradation of the matrix and the formation of by-products are evaluated by Fourier Transform Infrared (FTIR) and Raman spectroscopies. Secondly, surface morphological degradation, appearance of microcracks, and the phenomena of segregation or interfacial deterioration were evaluated by means of Field-Emission Scanning Electron Microscopy (FE-SEM). And finally, the optical and luminescent properties were evaluated by analyzing light decay curves (afterglow) in order to determine the net loss of emissive efficiency and the possible alterations in the mechanisms of entrapment and release of charge carriers.

The original materials were synthesized in a previous study 15 years ago and have remained exposed to the elements since then. Consequently, the present work focuses on the evaluation of the aging of the original formulations.

## 2. Materials and Methods

### 2.1. Preparation of Composites

The luminescent materials were synthesized from SrAl_2_O_4_ strontium aluminate powders, Eu^2+^, Dy^3+^, with a formula Sr_0.95_ Eu_0.062_ Dy_0.03_ Al_2_O_4_, with particle size of 11 microns, supplied by Sigma-Aldrich (St. Louis, MO, USA), a density of 4.2 g/cm^−3^, and their maximum luminescence at 575 nm. A thermoset polyester resin (Crystic 446 PALV, Scott Bader, Wellingborough, UK) was used as the matrix. The procedure followed consisted of direct mixing (40% luminescent pigment) and deaeration by stirring, followed by curing at 20 °C for 6 h, and post-curing at 40 °C for 16 h.

### 2.2. Natural Aging

**Figure 1:**
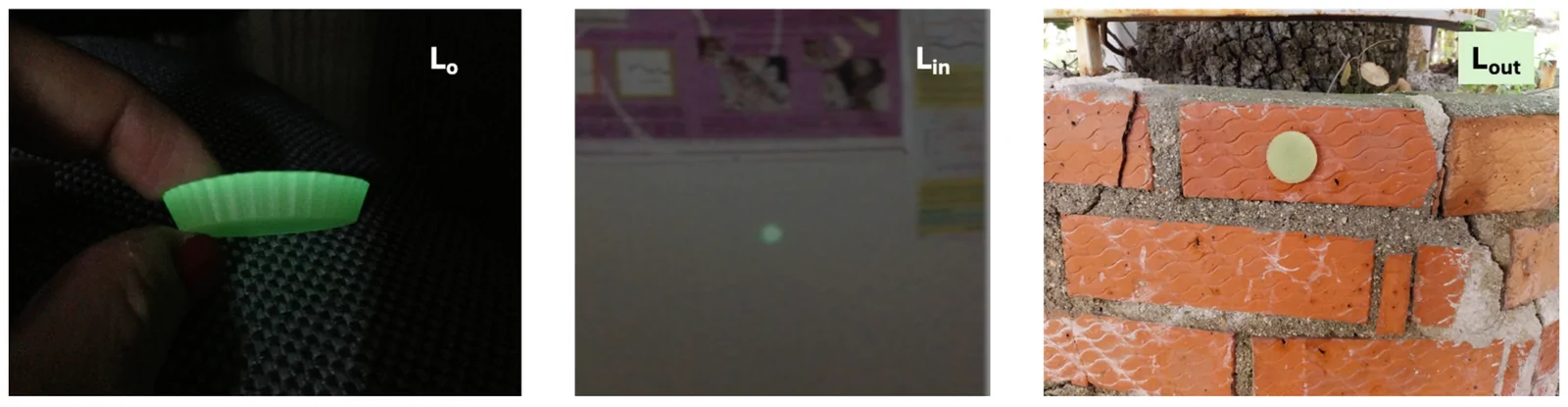
Specimens subjected to natural aging; L_0_ (original), L_in_ (indoor), L_out_ (outdoor).

To evaluate the degradation process, two different environments were chosen, one outdoors and located in Fresnedillas de la Oliva, Madrid, Spain, (40°29′10″ N and 4°10′27″ W) under real climatic conditions, and the other in an indoor room exposed to constant temperature and humidity (Figure 1). Three independent specimens were exhibited (approximately 5 mm thick) in every environment.

Indoor exposure was conducted in a standard office environment located in Extremadura (38°53′4″ N and 7°0′32″ W). Lighting was limited to standard artificial and natural office illumination (fluorescent/LED lights) during working hours, with the samples remaining in darkness during nights and weekends. The specimens were positioned away from exterior windows to prevent any direct exposure to solar UV radiation. Although continuous monitoring was not performed, typical indoor temperatures ranged approximately from 18 °C to 22 °C in winter and from 24 °C to 30 °C in summer (seasonally supported by HVAC heating and cooling systems). Relative humidity normally fluctuated between 30% and 60%, depending on the season.

**Figure 2:**
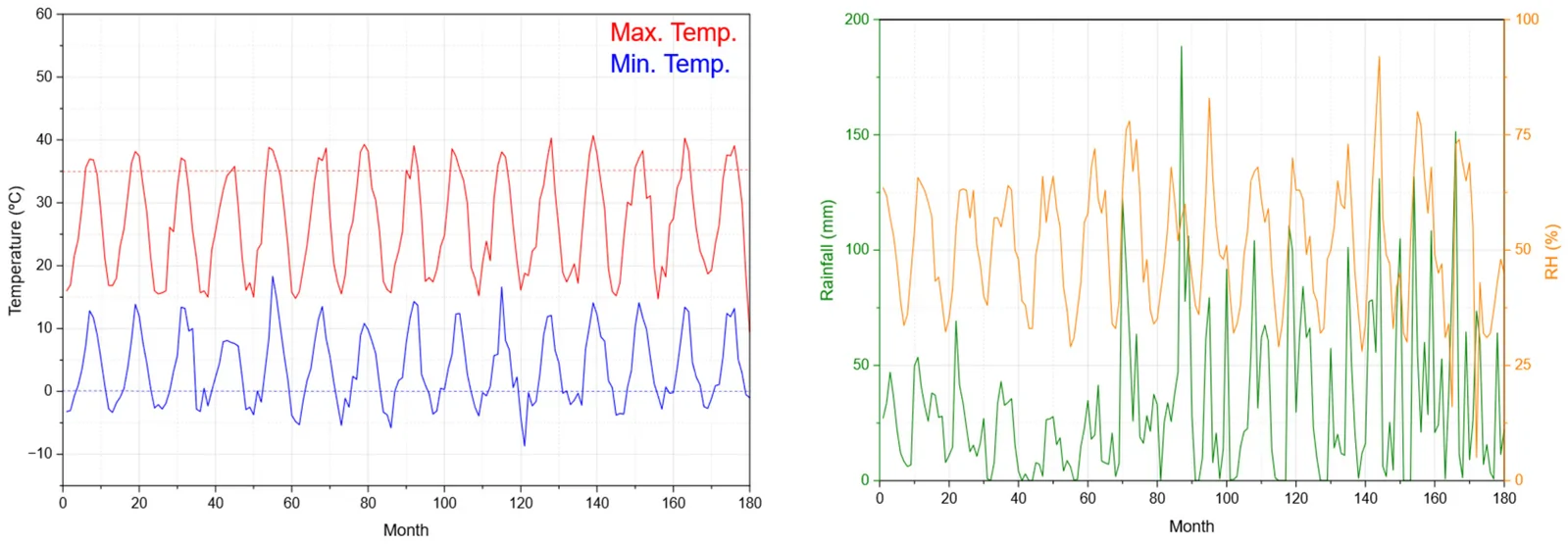
Evolution of meteorological parameters (maximum/minimum temperatures, precipitation and relative humidity) over the 15-year outdoor weathering exposure period.

The local climate profile of the exposure area during the 15-year trial is presented in Figure 2. The hydrothermal data (maximum and minimum temperatures, relative humidity and rainfall) were obtained from the official website of the Spanish State Meteorological Agency (AEMET) [26].

### 2.3. Specimen Preparation and Structural/Morphological Characterization

The original luminescent specimens (L_0_) were characterized directly after being removed from the mold. However, the indoor (L_in_) and outdoor (L_out_) aged specimens were gently cleaned to remove dust or adhered particles without altering their surface prior to characterization. All of them were studied by Fourier Transform Infrared (FTIR) and Raman spectroscopies, as well as FE-SEM.

The infrared spectra were obtained in a Perkin-Elmer Fourier transform infrared spectrophotometer Spectrum One model (PerkinElmer Inc., Shelton, WA, USA) using the Total Attenuated Reflectance (ATR) method with a resolution of 4 cm^−1^. Each spectrum corresponds to the average of 16 scans in the spectral region between 4000 and 600 cm^−1^.

The Raman spectra were performed on a Renishaw Raman spectrometer (Renishaw, Gloucestershire, Gloucester, UK) model inVia. The calibration of the equipment was carried out by recording the Raman spectrum of a monolithic silicon sample, which has its absorption band centered at 520 cm^−1^. The laser used was 785 nm.

The microscopic study was carried out under a HITACHI S-4700 FE-SEM Microscope (Hitachi Ltd., Tokyo, Japan), electron acceleration (20 KV), resolving power (70 Å), and current intensity (60 μA).

### 2.4. Flexural Strength and Microhardness

The evaluation of the mechanical properties of the synthesized strontium aluminate samples was carried out by means of three-point flexural strength tests. The mechanical test was carried out at room temperature using a universal Microtest testing machine, model EM2/200/FR (Microtest S.A., Madrid, Spain), equipped with a calibrated load cell of 1000 N and a speed of 5 mm/min. A minimum of 5 specimens were tested to obtain statistical representativeness. The breaking stress was calculated from the maximum load recorded and the specimen dimensions (width and thickness).

The microhardness tests were carried out on a Universal Indentation and Scratch Tester Model APEX-1 equipment (CETR, Campbell, CA, USA) with Berkovich tip on the surface of luminescent materials previously polished to a scratch-free metallographic finish, and with a roughness of less than 0.1 μm Ra.

### 2.5. Luminescent Characterization (Intensity, Persistence, and Color)

A 100 W bulb was used as the excitation source. The original (L_0_) and aged (L_in_ and L_out_) samples were placed inside a dark chamber to avoid any interference from ambient light. The bulb was positioned at a fixed distance from the sample and perpendicular to the beam, ensuring homogeneous illumination of the exposed surface, receiving an intensity of 1000 lm measured by a digital lux meter. The samples were irradiated at different saturation times (1, 2, 5 and 10 min).

Luminescence persistence (afterglow) was carried out through decay curves by continuously measuring the intensity of the emission immediately after turning off the excitation source (defined as t = 0 min), and up to 30 min. The half-life of the luminescent emission is determined by representing the experimental data of the emission intensity as a function of time. A Perkin-Elmer Lambda 40 UV-Vis spectrophotometer (PerkinElmer Inc., Shelton, WA, USA) at the wavelength of 575 nm was used to record the emission intensity as indicated by the product supplier.

Color changes on the surface of the aged luminescent materials were determined using a Konica-Minolta colorimeter model CM-2600d (Konica Minolta Inc., Tokyo, Japan). All measurements were made at room temperature, under identical geometric conditions and loading times to allow direct comparison of initial intensity, decay slope, and chromatic coordinates between different formulations or treatments of the aluminate.

### 2.6. Surface Recovery by Polishing

A rigorous evaluation of aging requires knowing if the loss of optical properties is due to intrinsic processes of photo-oxidation or a layer of dust, dirt, or other contaminants deposited on its surface over time. For this reason, the aged luminescent specimens were subjected to a mechanical polishing process using P4000 sandpaper (Illinois Tool Works Inc., Glenview, IL, USA), ensuring the homogeneous removal of the surface shell but without altering the structural integrity of the interior. The samples were then washed with distilled water and ethanol in an ultrasound bath for 10 min to remove polishing residues and dried under nitrogen flow. After polishing, structural analyses (FTIR, Raman), luminescence and colorimetry, were carried out to check if the original functionality was recovered.

## 3. Results and Discussions

### 3.1. Chemical and Structural Degradation (FTIR/Raman)

#### 3.1.1. FTIR Study

**Figure 3:**
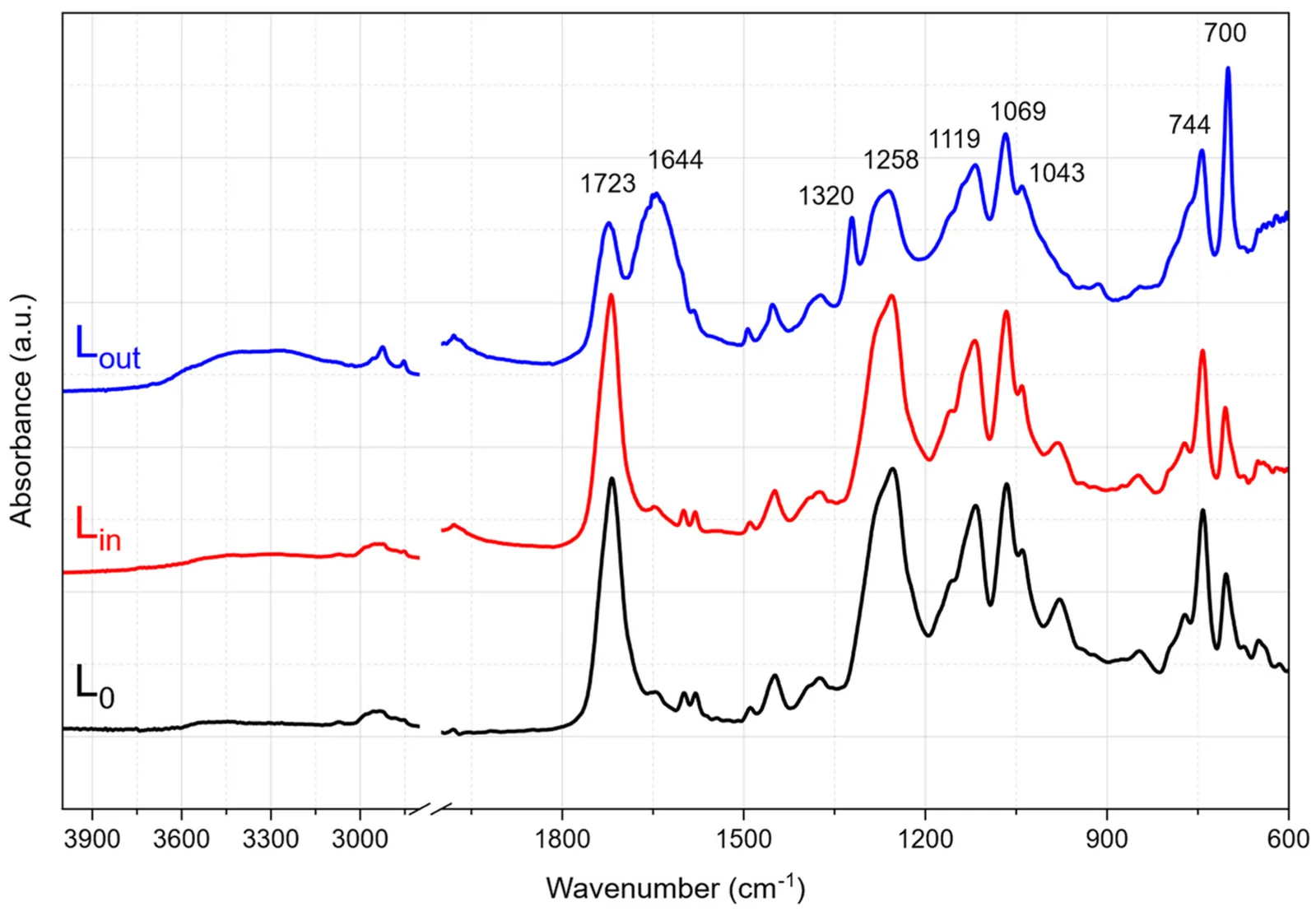
FTIR spectra of original and aged luminescent materials.

The surface chemical modification of luminescent composites before and after natural aging was analyzed by FTIR spectroscopy. Figure 3 shows the infrared spectra corresponding to all the samples studied (L_0_, L_in_ and L_out_) in the wavenumber range 4000–600 cm^−1^.

The IR spectrum of the original sample (L_0_) reveals the characteristic absorption bands of the structural matrix. In particular, the band located at 1723 cm^−1^ is consistent with the tension vibration of the carbonyl group (C=O), a typical signal of polyester resins and unsaturated polyesters [27]. Similarly, signals in the region 1120–1040 cm^−1^ agree with C–O tension vibrations of ester or ether bonds, since in polymers with C–O bonds this zone typically appears between 1300 and 1000 cm^−1^, and in polyester materials bands close to 1150, 1135, 1092, 1047, and 1042 cm^−1^ [28]. Also, the bands at 740 and 700 cm^−1^ can be assigned to deformations outside the plane of the C–H groups of the aromatic ring in accordance with the aromatic polymer literature, where these vibrations are generally located in the range 670–900 cm^−1^ [29].

In the spectrum of the sample aged under indoor conditions, an incipient band at 1644 cm^−1^ is identified, which can be attributed to the scissor bending, δ(HOH), of adsorbed water molecules, in agreement with FT-IR polymer studies showing that this signal increases with relative humidity and reflects the concentration of adsorbed water [30]. This band undergoes a notable increase in intensity and widening in the outside sample, which is consistent with an adsorption of ambient moisture and with the formation of more distributed and heterogeneous hydrogen bonding environments on the polymeric surface [30]. This interpretation is reinforced by the simultaneous increase in the broadband located at about 3500 cm^−1^, attributable to the O–H stretching of adsorbed water and/or surface hydroxyl groups, the intensity of which increases with humidification and with the environmental aging of weathered polymeric materials [31]. Overall, the behavior of both bands suggests that the outdoor exposure favored a greater hydration and surface hydroxylation of the resin than that observed in the sample aged indoors.

The band at 1320 cm^−1^ has been reported on the surface of stone materials exposed to outdoor environments and is attributed to calcium oxalate [32,33]. Its origin is linked to atmospheric pollution, but its presence also connects these films to the degradation of previous organic treatments [33,34]. On stone surfaces, bacteria, fungi, algae, and lichens form biofilms, whose metabolic activity favors biodeterioration and biomineralization processes. Various lines of evidence indicate that these microorganisms can produce oxalic acid and other organic acids capable of reacting with calcium phases of the substrate to precipitate oxalates, including whewellite and weddellite [35,36,37]. Nevertheless, the atmospheric contribution should not be excluded, as it has also been proposed that a fraction of the oxalate may originate from aerosols or from reactions between atmospheric pollutants, dust, and carbonate surfaces [32,33]. However, this pathway is typically considered complementary and less proven than the microbial route [37].

#### 3.1.2. Raman Study

**Figure 4:**
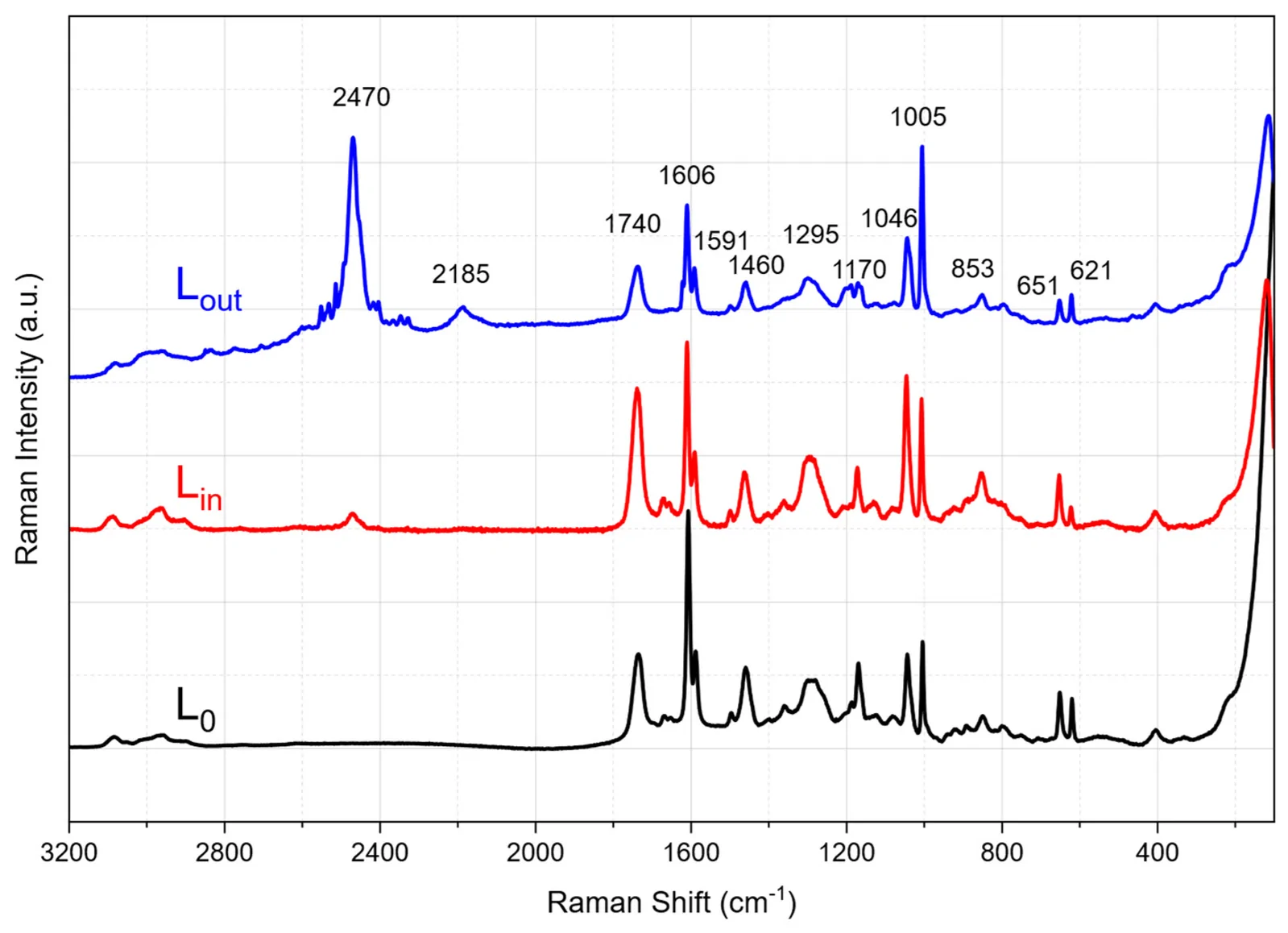
Raman spectra of original and aged luminescent materials.

Figure 4 compares the Raman spectra of the original resin and of the aged samples indoors and outdoors. The spectrum of the original sample shows the characteristic bands of a polyester resin: 1740 cm^−1^ (C=O), 1606 and 1591 cm^−1^ (aromatic ring), 1460 cm^−1^ (saturated C–H flexion), 1170 cm^−1^ (ester group), 1046 cm^−1^ (C–O tension), 1005 cm^−1^ (mono benzene and meta-substituted), and 651–620 cm^−1^ (C–H para-substituted) [38].

As observed in the IR study, the sample aged indoors does not show any noticeable structural changes, i.e., in the absence of intense photochemical conditions, polyesters can maintain high overall chemical stability, although they present small changes in the conformational distribution or in the relative intensity of bands. Thus, the change in the relative intensity of the 1046 and 1005 cm^−1^ bands could be interpreted as a local reorganization of C-O/aromatic contributions rather than as a major structural transformation of the polymer. Similarly, the presence of a weak band located at 2470 cm^−1^ is compatible with the incipient appearance of new surface species, rather than with a massive degradation of the matrix [28].

On the other hand, the sample aged outdoors shows more marked changes, consistent with the fact that weathering accelerates surface photochemistry, the cleavage of ester bonds, and the generation of new chromophores or oxidation products in polyesters exposed to light, oxygen, and water. The strong increase in the band located at 2470 cm^−1^ suggests that this signal responds to an adhered surface component or a shell developed during exposure, rather than to a main vibration of the main resin. This interpretation is also consistent with the fact that external aging favors mineralogical differences between protected and exposed areas, as well as the stratified accumulation of complex surface films [28]. The presence of a small band at 2185 cm^−1^ is directly associated with external environmental degradation, since in the 2100–2250 cm^−1^ Raman region, the polymer and aluminate do not present any vibration. As extreme photochemical degradation has not been observed in IR but the presence of calcium oxalate CaC_2_O_4_ was detected, it is logical to consider that it crystallizes and forms complex crusts in the open air, and this vibration is due to secondary combination bands [39].

### 3.2. Characterization of the Surface Morphological Changes (FE-SEM)

The small chemical and structural changes observed in aged materials are barely reflected in the microstructure. Although the literature reports localized mass losses within the polyester matrix when the materials have been exposed to weathering [40,41], no significant surface changes have been found in the aged samples with respect to the original material.

**Figure 5:**
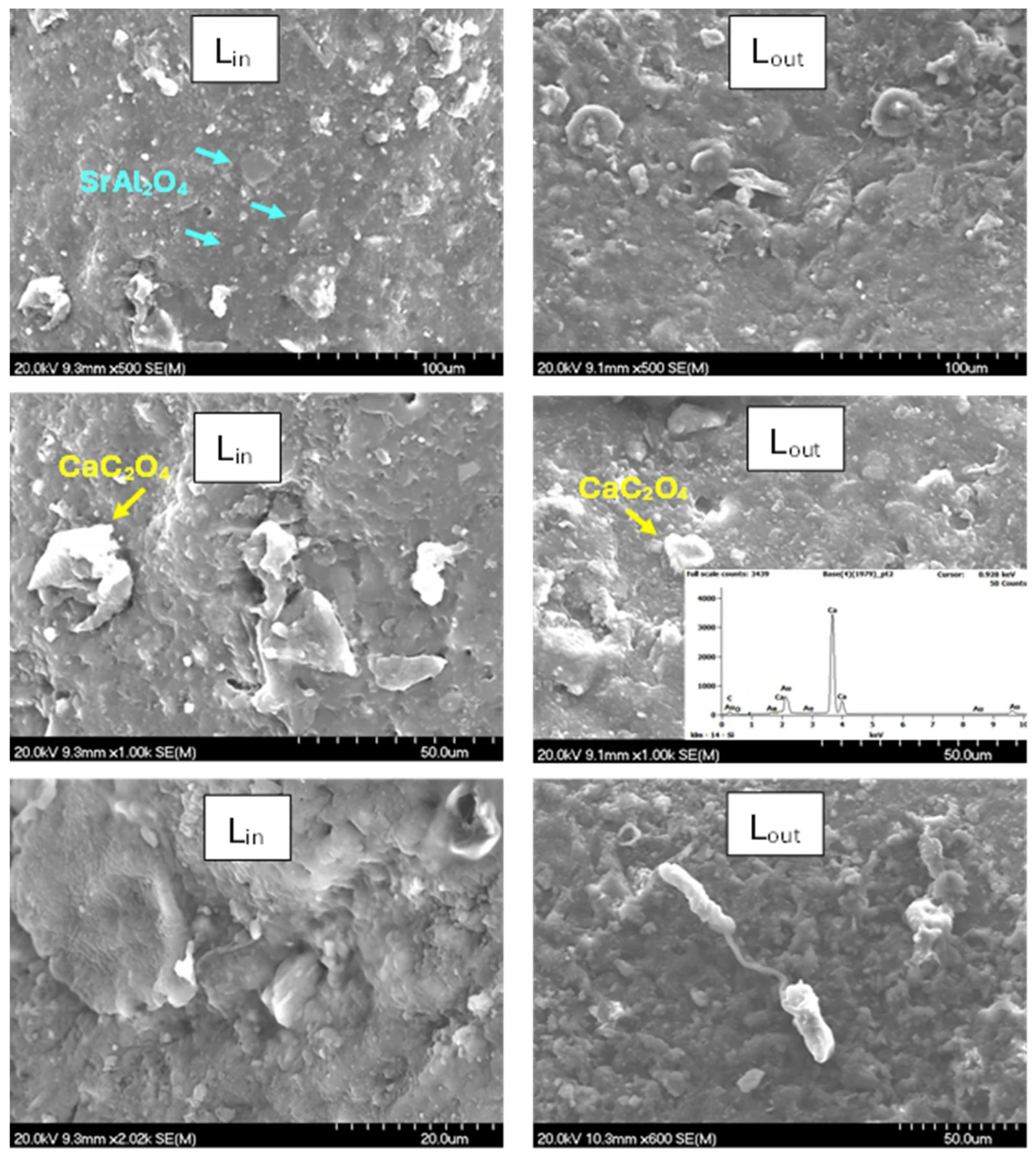
FE-SEM micrographs of L_in_ and L_out_ samples at different magnifications.

Figure 5 shows the FE-SEM photographs of the surface of the luminescent materials aged indoors and outdoors at different magnifications. Both surfaces are globally smooth, compact and continuous, with no evidence of massive delamination, cracks or signs of significant deterioration over the years. Even the detachment of the strontium aluminate grains is not observed, which is consistent with the protective behavior played by the polymeric matrix that has been described in the literature for this type of composites [14].

The generation of cracks or microcracks has been widely studied in composites subjected to thermocycling [42,43], caused by the difference between the coefficients of thermal expansion of the polyester matrix and the inorganic filler. However, in the studied materials, no microcracks are observed around the strontium aluminate particles, despite the fact that the sample aged outdoors was exposed year after year to harsh winters and very hot summers, so it was subjected to numerous cycles of thermal expansion/compression.

### 3.3. Impact of Natural Aging on Mechanical Properties

#### 3.3.1. Flexural Strength

**Figure 6:**
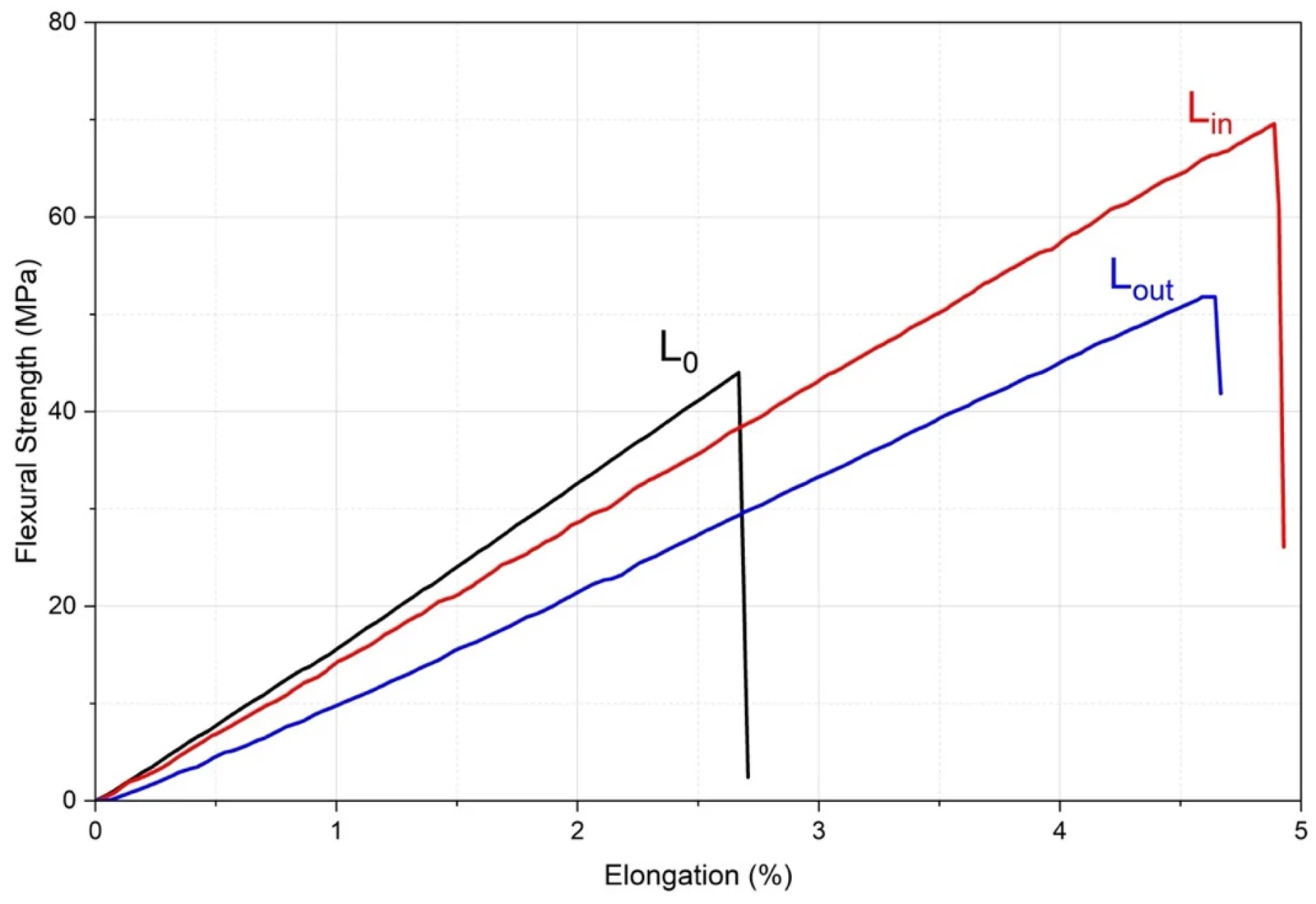
Flexural stress–strain curves obtained from three-point bending tests curves of the studied luminescent materials.

The stress–strain curves corresponding to the bending test of the original sample and the aged ones (Figure 6) show a linear behavior throughout the analysis interval, breaking catastrophically in a single stage, characteristic of brittle materials.

The literature has shown that the mechanical response of polymeric composites is closely linked to their degree of aging [44,45]. Consequently, it would be reasonable to anticipate that the deterioration of mechanical properties in bending and microhardness would constitute a direct indicator of structural stability and fatigue resistance during life service. However, in this study the tensile strength (MOR) of the materials subjected to natural aging increases, while the modulus of elasticity (Young modulus) remains practically unchanged (Table 1). This decoupled response has been attributed to the fact that MOR and Young’s modulus are governed by distinct degradation mechanisms, in line with what has been previously described for other polymeric matrix systems [45,46].

The increase in tensile strength observed in indoor aged specimens (from 44 to 72 MPa) can be related to the post-curing and densification of the matrix and/or to an internal rearrangement that leads to the partial closure of microdefects (pores), favoring greater cohesion and resistance and reducing the trend of cracking under in-service loads, without appreciably modifying the overall elastic stiffness (3.9–3.6 GPa). The sample exposed outdoors follows a similar trend, although less marked, due to the additional deterioration associated with water and UV radiation, which can induce changes in the crystallinity of the polymer phase, hardening the microstructure and raising the MOR value [47,48]. In fact, numerous studies report this type of decoupled behavior, in which Young’s modulus is hardly affected by aging, despite appreciable variations in flexural strength [49,50].

#### 3.3.2. Microhardness

The observed increase in microhardness after the aging process (Table 1) suggests the existence of a post-curing phenomenon and a consequent increase in the crosslinking density of the surface zone due to the progressive elimination of residual monomers [51,52].

During aging, the combined action of time, temperature and UV radiation is capable of promoting additional matrix cross-linking, although this mechanism coexists in parallel with competing processes of photo-oxidation and polymer chain cleavage [53,54].

The fact that the weathered sample exhibits a slightly higher microhardness than the indoor weathered sample (0.37 GPa vs. 0.33 GPa) supports the formation of a highly cross-linked surface layer due to direct exposure to environmental factors, especially UV radiation, thermal shock, and oxygen [55,56]. However, in the studied samples, this localized hardening does not imply an improvement in the overall mechanical integrity of the material, coinciding with other studies that found that environmental aging can induce marked surface deterioration, increase brittleness, and reduce flexural strength, even when the hardness at the periphery remains high or increasing [57,58].

### 3.4. Light Performance and Colorimetry

#### 3.4.1. Decay Mechanisms

**Figure 7:**
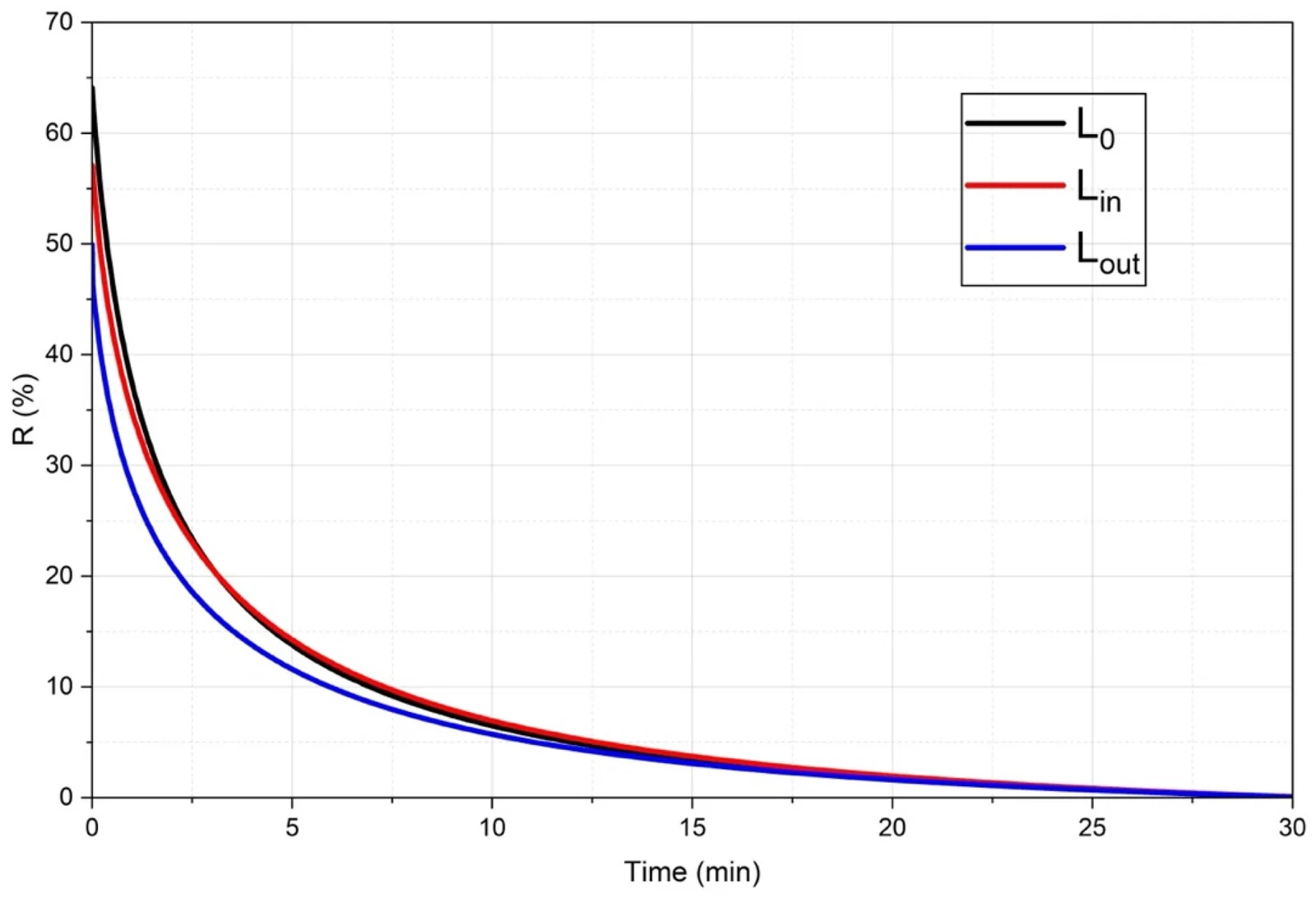
Time-dependent phosphorescence intensity decay of L_0_, L_in_, and L_out_ after 10 min of photo-stimulation.

The decay curves (Figure 7) show that in these materials, the aging hardly modifies the light attenuation kinetics, showing a very rapid drop in the first minutes followed by a slower decay. These curves are typical of luminescent strontium aluminate materials with trap centers of varying depths, with defects controlling decay and afterglow [20,59,60].

The fit of the intensities obtained from the decay curves to a second-order exponential function [61] provides the effective lifetimes, t_1_ and t_2_, which indicate the speed at which the light signal is turned off [62,63]. Table 2 summarizes the parameters obtained from the setting for an excitation time of 10 min. The similarity found for t_1_ and t_2_ in the aged samples, with respect to the original one, demonstrate that the luminescent materials maintain their internal consistency and stability against adverse external conditions, with reproducible kinetics after 15 years of life in service. The t_1_ does not vary from the original sample (1.12 min) to the aged samples (1.05–1.03 min), indicating that the processes of rapid de-excitation associated with shallow traps or emitting centers (Eu^2+^) have hardly been affected by aging in the polymer [64,65]. The small increases in t_2_ (from 6.97 to 7.31–7.34 min) suggest a conservation and even small stabilization of the deep traps responsible for the afterglow, consistent with the high chemical stability of SrAl_2_O_4_:Eu^2+^,Dy^3+^ in suitable matrices [66,67].

However, the light intensity is affected by aging, reducing by 11% in the indoor sample and almost twice in the outdoor sample. Havasi et al. [68] noted that the stability of the time constants (t_1_, t_2_) after potential decades of use suggests that the eventual loss of brightness observed in real applications is mainly due to the decrease in the number of emitting centers or surface changes, rather than to a modification of the decay mechanism. Studies in other polymeric luminescent systems show that weathering usually affects intensity primarily, while time constants can remain relatively stable if the array protects the luminophore phase [69].

#### 3.4.2. Colorimetry

The evolution of color after 15 years of life in service was analyzed using CIELAB coordinates, since it is the usual method to study aging in strontium aluminate materials [70,71]. Table 3 shows these values, where L* represents the light from white to black, a* is the red (+)/green (−) chromatic trend, and b* the blue (−)/yellow (+) trend. The drop of eight units of L* in the indoor sample and three in the outdoor sample indicates a darkening in both environments, although more pronounced indoors. This pattern does not necessarily indicate greater aging, but rather that the environmental conditions to which they have been exposed have affected their final appearance differently [70].

The small change shown in a* suggests a shift towards greener tones, consistent with the usual chromatic identity of SrAl_2_O_4_: Eu^2+^,Dy^3+^, whose typical emission around 515–575 nm reported for photoluminescent polyesters with SrAl_2_O_4_: Eu^2+^,Dy^3+^, and visible perception, are described in the literature as green, green-yellow or yellow-greenish, depending on the composition, microstructure, and state of excitation [70,72]. The clearest indication of visible aging is the change observed in b*, which shows more intense yellowing outdoors, corroborating the results obtained from FTIR and Raman.

The observed color changes, darkening in the indoor sample and yellowing in the outdoor one, do not imply a total loss of their optical functionality since they have preserved their overall visual response reasonably well without total failure in the appearance of the material (luminescence continues).

### 3.5. Surface Restoration by Polishing

#### 3.5.1. Structural Recovery (IR, Raman)

**Figure 8:**
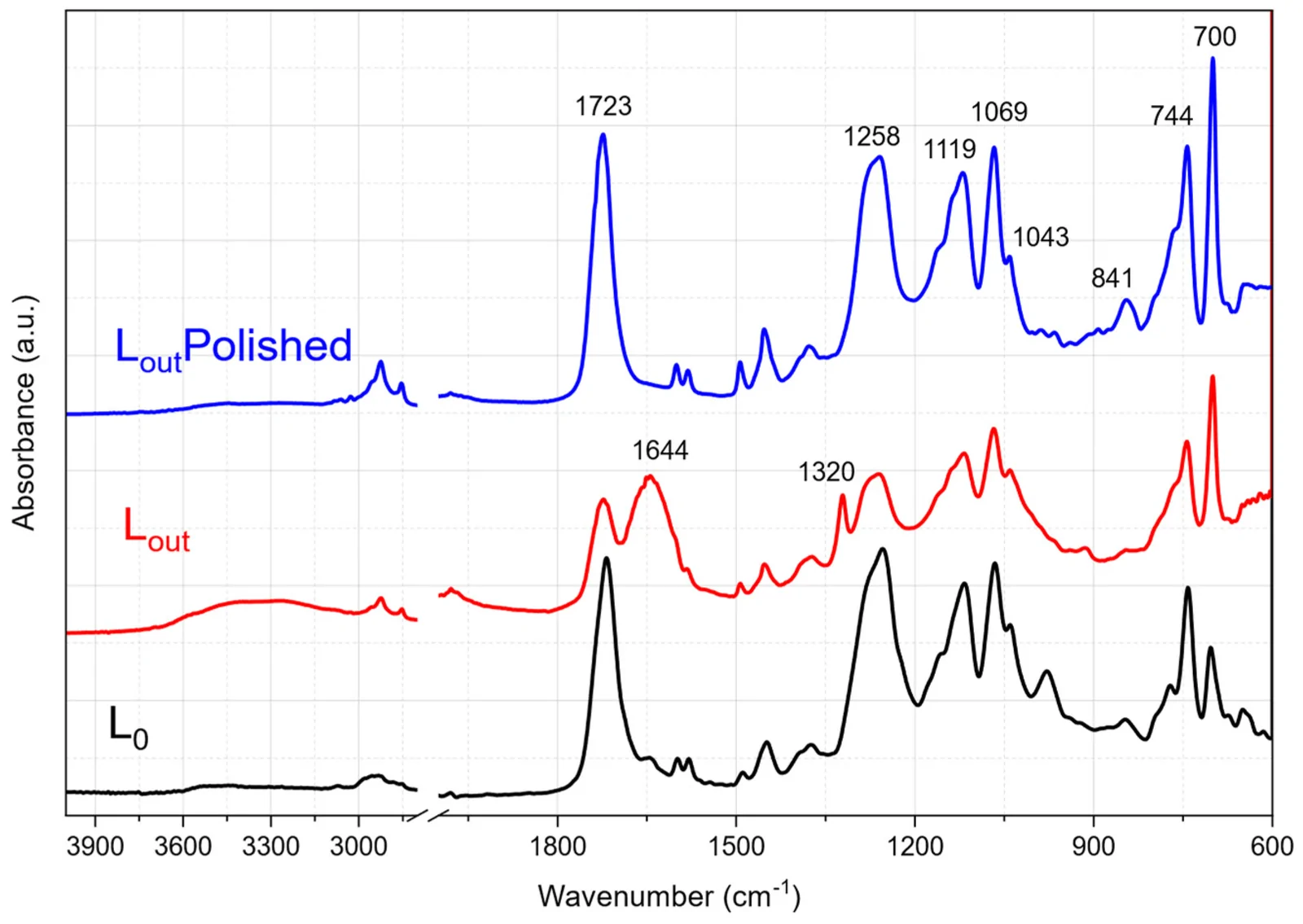
FTIR spectra of outdoor aged luminescent, polished and unpolished.

The FTIR spectra of the polished surface (Figure 8) show an almost complete match with the spectrum of the original luminescent sample (L_0_), along with the decrease or disappearance of bands attributable to exogenous surface contamination. FTIR allows us to distinguish surface chemical changes, but its interpretation requires separating signals of polymer oxidation from signals due to adsorbed contaminants or surface biomass [29,73].

The combined reduction in the intensities of the bands located at 3500 and 1644 cm^−1^ after polishing indicates that these signals do not evidence an intrinsic degradation caused by UV radiation but are compatible with surface species retained in the layer of adsorbed dust or moisture. This type of ambiguity is a known problem in the FTIR of oxidized polymers, because O–H, C–O, and C=O bands can come from both oxidation of the material and surface biological or atmospheric contamination [73].

The IR study showed the presence of calcium oxalate by the band at 1320 cm^−1^ due to the symmetrical stretching of the carboxyl group O-C=O. The disappearance after polishing is compatible with the removal of surface deposits rather than with a structural transformation of the polymer [74,75]. As mentioned above, this compound is frequently detected in materials subjected to weathering, either by reactions between atmospheric species and deposited mineral particles, or by the activity of microorganisms capable of generating surface biofilms.

**Figure 9:**
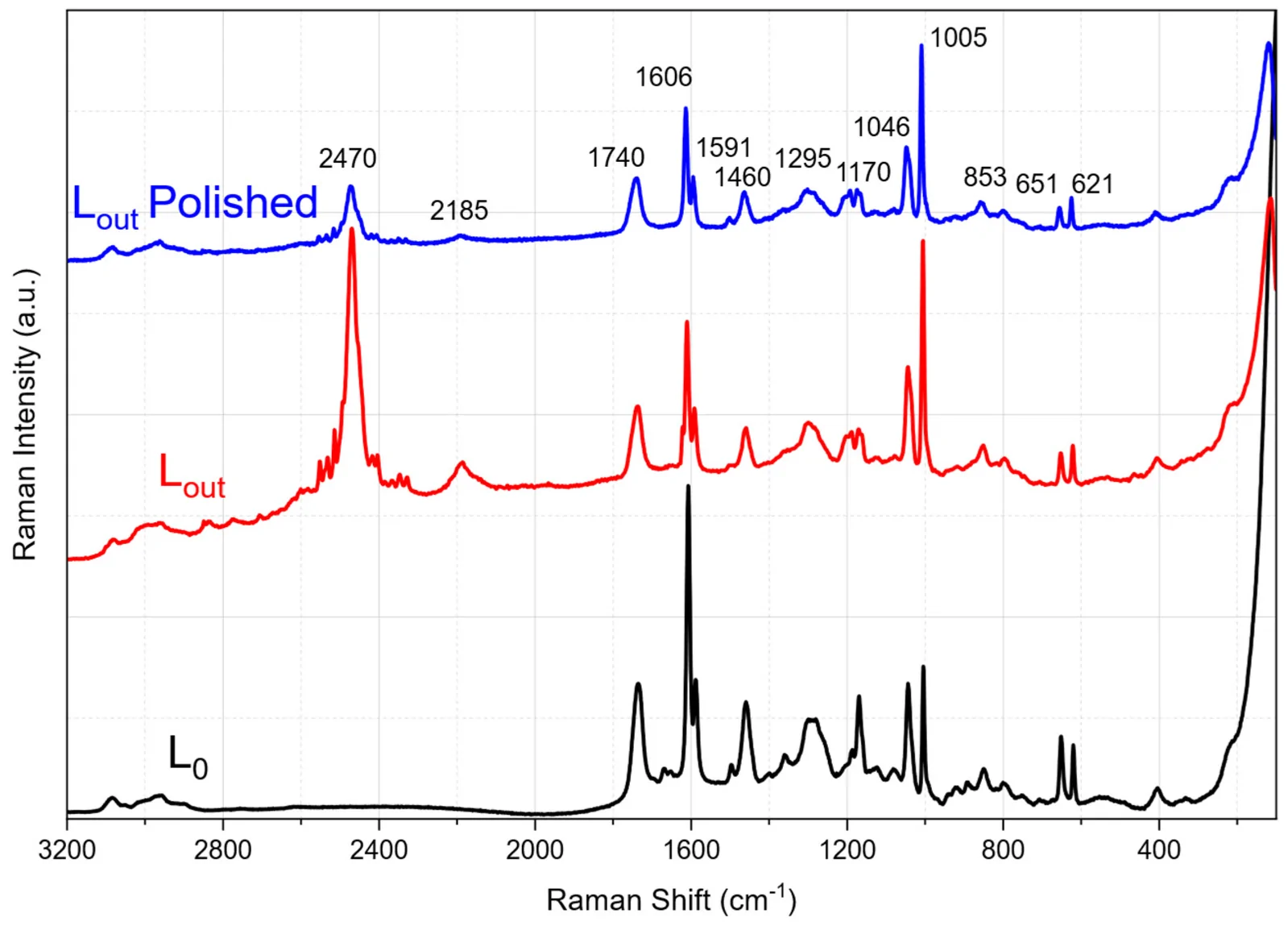
Raman spectra of outdoor aged luminescent, polished and unpolished.

The Raman study (Figure 9) reveals that after polishing, the signals associated with exogenous contamination disappear, demonstrating that chemical degradation and dust accumulation were strictly confined to a surface micrometer layer, keeping the internal chemical structure of the matrix intact [28].

To further investigate the composition and origin of the 15-year outdoor fouling layer, Energy-Dispersive X-ray Spectroscopy (EDS) was conducted on the polished and unpolished surfaces. Comparative EDX analysis elucidated the nature of the degradation layer and confirmed the effectiveness of the surface restoration. Unpolished surface exhibited surface-identified signals of Ca and Fe alongside the matrix elements (O, Al, and Sr), demonstrating the accumulation of external atmospheric contaminants such as mineral dust and urban/industrial airborne particulates (Figure S1). Conversely, following the polishing procedure, the Ca and Fe peaks completely disappeared (Figure S2), leaving only the constituent elements of the strontium aluminate substrate (O, Al, and Sr) and a minor carbon (C) signal, likely arising from adventitious carbon or sample handling (trichome, cell, or vegetable fiber/cotton). The complete elimination of exogenous contaminants (Ca and Fe) upon mechanical removal of the outer layer confirms that the fouling crust forms an external, reversible physical barrier, serving as a major mechanism responsible for the optical attenuation after 15 years of outdoor exposure. The elemental EDS mapping of both polished and unpolished surface crust are provided in the Supplementary Materials (Figures S1 and S2).

#### 3.5.2. Recovery of Light and Color Properties

Table 4 shows the reflectance values of the samples aged indoors and outdoors after polishing, as well as of the original sample. The decrease in reflectance observed in aged samples (from 64% in the original sample to 57% and 49% indoors or outdoors, respectively) is a clear indication that environmental exposure significantly deteriorates the optical behavior of the polymer. This reduction is more pronounced outdoors, as the specimens were exposed to an unprotected external environment and directly exposed to dust, dirt or other environmental contaminants. The literature indicates that an outdoor environment offers favorable conditions for a more severe optical loss than indoors because deposition depends on local factors such as atmospheric pollution, humidity, wind, rain, roughness and surface orientation [76], and that this reduction is directly proportional to the amount of the deposit [77]. Profilometric measurements demonstrate that polishing selectively removed a thin, heterogeneous environmental crust of approximately 0.4 μm to 11.2 μm lodged in the surface relief, preserving the underlying polymer matrix (Tables S1 and S2 and Figures S3 and S4 included in Supplementary Materials).

The recovery of reflectance after polishing, 7% in the indoor sample and 10% in the outdoor sample, suggests that the dirt adhered to the surface acts in both cases as a physical filter that blocks both the excitation and the luminescent emission of the active centers of Eu^2+^ and Dy^3+^ (optical screening effect). The fact that the inner pigment remains active after polishing ratifies the protective barrier that the polyester matrix uses against ambient humidity, preventing its hydrolysis.

The incomplete recovery in the outdoor sample (reflectance remains below the original value after polishing) suggests that dirtying alone does not explain the loss of functionality, so that outdoor exposure will have incorporated some additional component of degradation associated with UV radiation, moisture, gaseous contaminants, and/or surface morphological changes. It follows that a significant fraction of optical deterioration is reversible by the removal of one micron of the superficial layer rather than by deep structural damage [78].

The colorimetric analysis supports that the ambient dust causes a certain chromatic deviation towards values of greater yellowing and a loss of luminosity. The colorimetric results (Table 4) indicate that the polishing produced measurable changes in the CIELAB coordinates of the samples exhibited indoors and outdoors. In the interior sample, the increase in L* from 37.7 to 39.3 suggests an optically brighter surface after polishing, in agreement with studies showing that the reduction in surface roughness increases light reflection and raises apparent luminosity [79,80]. The variation in a* towards more negative values (from −3.7 to −4.45) suggests a slight accentuation of the green component while the slight increase in b* (from 11.1 to 11.4) indicates a minimal variation towards yellow. However, these chromatic modifications are small compared to the dominant change in luminosity, which coincides with the evidence that surface texture especially affects the L* value [79].

The increase in L* (42 to 43), accompanied by the decrease in b* (14.8 to 13.9), suggests that polishing favored a partial recovery of luminosity. This finding is consistent with studies showing that a surface treatment at the micron level can reverse perceptible colorimetric changes and simultaneously improve roughness and optical transmittance [80], even though some of the color change comes from within the composite [81,82].

Our initial hypothesis was based on the widely reported intrinsic degradation mechanisms of these materials, such as yellowing, oxidation, and cracking upon prolonged exposure. However, the experimental results revealed that extrinsic factors, specifically, the accumulation of a surface dirt layer (soiling), acted as the dominant and fastest driver for luminosity loss, surpassing the impact of the expected chemical degradation. This discrepancy constitutes one of the most relevant findings of this work, highlighting that under real-world exposure over 15 years, physical soiling operates as the primary limiter of optical performance before severe structural or chemical failure occurs. Consequently, this unexpected outcome fundamentally shifts how service-life predictions for outdoor luminescent materials should be approached; the “end-of-life” criterion regarding optical performance is not solely dictated by irreversible chemical failure, but is strongly influenced by surface soiling, which could potentially be reversed through routine maintenance or cleaning cycles.

## 4. Conclusions

From the obtained results, it can be concluded that the luminescent materials, analyzed after their natural aging in indoor and outdoor conditions, satisfactorily preserve their structural, mechanical, and optical functionality properties without evidence of general failure.

Both mechanical strength and hardness increase after aging, although the modulus of elasticity remains constant. This means that the passage of time does not compromise the overall rigidity of the materials, although it does seem to favor certain resistant parameters, probably as a result of physical or structural readjustments of the polymer matrix.

The reduction in light intensity and gradual loss of brightness are expected results after a decade and a half of exposure to the elements, but this does not imply the disappearance of the luminescent capacity, but a partial decrease in its performance, compatible with the preservation of the essential optical performance of the system. However, the similarity of the t_1_ and t_2_ kinetic parameters between the original sample and the aged ones suggests that the mechanisms of energy trapping and release linked to europium (Eu) ions remain substantially unchanged after aging. This is a particularly relevant result, as it demonstrates that the functional core responsible for persistent luminescence has not been significantly altered by the time or by the environmental conditions of exposure.

The sample aged indoors has a greater darkening, although polishing allows the luminosity to be partially restored. Similarly, the sample aged outdoors shows more intense yellowing, which also does not completely disappear after polishing. These results reinforce the idea that the chromatic alteration of the material induced by aging is not limited exclusively to the surface layer, but also extends to deeper areas of the matrix.

With the polishing of the surface, it has been possible to separate the “noise” of contamination from the actual damage to the luminescent polyester, since the loss of brightness and clarity is not exclusively due to an intrinsic degradation of the polyester resin, but also to the presence of a surface-contaminating shell layer. However, the incomplete optical recovery in the outdoor sample evidences the coexistence with intrinsic irreversible chemical transformations linked to exposure to weathering.

Overall, the findings show that, despite the modifications induced by 15 years of natural aging, these materials maintain their structural integrity, mechanical response, and optical-luminescent functionality. Therefore, it can be said that the system was adequately designed, both in the choice of matrix and in the concentration of pigment selected, and that these materials have sufficient durability to anticipate an additional extension of their useful life in service.

## Figures and Tables

**Table 1:** Data of tensile strength, modulus of elasticity and microhardness.

	MOR (MPa)	Young (GPa)	Microhardness (GPa)
L_0_	44 ± 2	3.9 ± 0.1	0.14 ± 0.01
L_in_	72 ± 4	3.6 ± 0.1	0.33 ± 0.01
L_out_	54 ± 3	3.8 ± 0.1	0.37 ± 0.01

**Table 2:** Parameters obtained from exponential fit.

	I_0_	A_1_	t_1_ (min)	A_2_	t_2_ (min)
L_0_	64	33.98	1.121 ± 0.008	27.62	6.97 ± 0.03
L_in_	57	26.97	1.105 ± 0.008	27.93	7.31 ± 0.03
L_out_	50	22.35	1.034 ± 0.008	22.84	7.34 ± 0.04

**Table 3:** CIELAB color coordinates of luminescent aged materials.

Coordinates	Polyester Resin	L_in_	L_out_
L*	46.3	37.71	42.63
a*	−3.10	−3.73	−3.78
b*	9.91	11.17	14.86

**Table 4:** CIELAB color coordinates of unpolished and polished luminescent aged materials.

Coordinates	Polyester Resin	Unpolished		Polished	
		L_in_	L_out_	L_in_	L_out_
L*	46.3	37.71	42.63	39.3	43.5
a*	−3.10	−3.73	−3.78	−4.45	−3.8
b*	9.91	11.17	14.86	11.4	13.9

## Data Availability

The original contributions presented in this study are included in the article/Supplementary Materials. Further inquiries can be directed to the corresponding author.

## Supplementary Materials

The following supporting information can be downloaded at: https://www.mdpi.com/article/10.3390/polym18161949/s1.

## References

[1] Li Y., Gecevicius M., Qiu J. (2016). Long Persistent Phosphors—From Fundamentals to Applications. Chem. Soc. Rev..

[2] Van Den Eeckhout K., Bos A.J.J., Poelman D., Smet P.F. (2013). Revealing Trap Depth Distributions in Persistent Phosphors. Phys. Rev. B.

[3] Yang L., Gai S., Ding H., Yang D., Feng L., Yang P. (2023). Recent Progress in Inorganic Afterglow Materials: Mechanisms, Persistent Luminescent Properties, Modulating Methods, and Bioimaging Applications. Adv. Opt. Mater..

[4] Parayil R.T., Gupta S.K., Mohapatra M. (2025). A Review on Defect Engineered NIR Persistent Luminescence through Transition Metal Ion (Cr, Mn, Fe and Ni) Doping: Wider Perspective Covering Synthesis, Characterization, Fundamentals and Applications. Coord. Chem. Rev..

[5] Van Der Heggen D., Vandenberghe D., Moayed N.K., De Grave J., Smet P.F., Joos J.J. (2020). The Almost Hidden Role of Deep Traps When Measuring Afterglow and Thermoluminescence of Persistent Phosphors. J. Lumin..

[6] Liang L., Chen J., Shao K., Qin X., Pan Z., Liu X. (2023). Controlling Persistent Luminescence in Nanocrystalline Phosphors. Nat. Mater..

[7] Gao P., Liu Q., Wu J., Jing J., Zhang W., Zhang J., Jiang T., Wang J., Qi Y., Li Z. (2023). Enhanced Fluorescence Characteristics of SrAl_2_O_4_: Eu^2+^, Dy^3+^ Phosphor by Co-Doping Gd^3+^ and Anti-Counterfeiting Application. Nanomaterials.

[8] Peng T., Huajun L., Yang H., Yan C. (2004). Synthesis of SrAl_2_O_4_:Eu, Dy Phosphor Nanometer Powders by Sol–Gel Processes and Its Optical Properties. Mater. Chem. Phys..

[9] Rojas-Hernandez R.E., Rubio-Marcos F., Rodriguez M.Á., Fernandez J.F. (2018). Long Lasting Phosphors: SrAl_2_O_4_:Eu, Dy as the Most Studied Material. Renew. Sustain. Energy Rev..

[10] Gültekin S., Yıldırım S., Yılmaz O., Keskin İ.Ç., Katı M.İ., Çelik E. (2019). Structural and Optical Properties of SrAl_2_O_4_: Eu^2+^/Dy^3+^ Phosphors Synthesized by Flame Spray Pyrolysis Technique. J. Lumin..

[11] Li Z., Hao S., Ji W., Hao L., Yin L., Xu X., Agathopoulos S. (2021). Mechanism of Long Afterglow in SrAl_2_O_4_:Eu Phosphors. Ceram. Int..

[12] Qiu C., Chen J., Cui J., Geng X., Wang H., Bo S. (2007). Synthesis of Long Afterglow Phosphors Doped B SrA1_2_O_4_:Eu^2+^, Dy^3+^ and Its Luminescent Properties. J. Rare Earths.

[13] Maraveas C., Kyrtopoulos I.V., Arvanitis K.G., Bartzanas T. (2024). The Aging of Polymers under Electromagnetic Radiation. Polymers.

[14] Poulose A.M., Shaikh H., Anis A., Alhamidi A., Kumar N.S., Elnour A.Y., Al-Zahrani S.M. (2022). Long Persistent Luminescent HDPE Composites with Strontium Aluminate and Their Phosphorescence, Thermal, Mechanical, and Rheological Characteristics. Materials.

[15] Pinochet N., Pirot-Berson L., Couderc R., Therias S. (2024). UV LED Ageing of Polymers for PV Cell Encapsulation. npj Mater. Degrad..

[16] Tubio C.R., Seoane-Rivero R., Neira S., Benito V., Zubieta K.G., Lanceros-Mendez S. (2022). Fiber-Reinforced Polyester Composites with Photoluminescence Sensing Capabilities for UV Degradation Monitoring. Polymers.

[17] Prudnikau A., Shiman D.I., Ksendzov E., Harwell J., Bolotina E.A., Nikishau P.A., Kostjuk S.V., Samuel I.D.W., Lesnyak V. (2021). Design of Cross-Linked Polyisobutylene Matrix for Efficient Encapsulation of Quantum Dots. Nanoscale Adv..

[18] Liu Y., Chen T., Jin Z., Li M., Zhang D., Duan L., Zhao Z., Wang C. (2022). Tough, Stable and Self-Healing Luminescent Perovskite-Polymer Matrix Applicable to All Harsh Aquatic Environments. Nat. Commun..

[19] Jiang G., Guhrenz C., Kirch A., Sonntag L., Bauer C., Fan X., Wang J., Reineke S., Gaponik N., Eychmüller A. (2019). Highly Luminescent and Water-Resistant CsPbBr_3_ –CsPb_2_ Br_5_ Perovskite Nanocrystals Coordinated with Partially Hydrolyzed Poly(Methyl Methacrylate) and Polyethylenimine. ACS Nano.

[20] Poulose A.M., Shaikh H., Anis A., Alhamidi A., Kumar N.S., Elnour A.Y., Al-Zahrani S.M. (2022). Effect of Compatibilizer on the Persistent Luminescence of Polypropylene/Strontium Aluminate Composites. Polymers.

[21] Rizzo-Sierra J.A., Montoya-Santiyanes L.A., Isaza C., Anaya K., Ramirez-Gutierrez C.F., Zavala De Paz J.P. (2025). Multi-Statistical Pragmatic Framework to Study UV Exposure Effects via VIS Reflectance in Automotive Polymer Components. Polymers.

[22] Nzimande M.C., Mtibe A., Tichapondwa S., John M.J. (2024). A Review of Weathering Studies in Plastics and Biocomposites—Effects on Mechanical Properties and Emissions of Volatile Organic Compounds (VOCs). Polymers.

[23] Salim M.S., Ariawan D., Ahmad Rasyid M.F., Mat Taib R., Ahmad Thirmizir M.Z., Mohd Ishak Z.A. (2020). Accelerated Weathering and Water Absorption Behavior of Kenaf Fiber Reinforced Acrylic Based Polyester Composites. Front. Mater..

[24] Elkori R., Lamarti A., El Had K., Hachim A., Yamari I. (2024). Evaluation of the Impact of Natural Weathering on the Properties of High-density Polyethylene Bottles by Experimental Approach. Polym. Eng. Sci..

[25] Xu J.J., Zhang Y.H., Rutqvist J., Hu M.S., Wang Z.Z., Tang X.H. (2023). Thermally Induced Microcracks in Granite and Their Effect on the Macroscale Mechanical Behavior. JGR Solid Earth.

[26] Meteorología A.E. de Agencia Estatal de Meteorología—AEMET. Gobierno de España. https://www.aemet.es/es/portada.

[27] Jero D., Wärnheim A., Caussé N., LeBozec N., Pébère N., Persson D., Thierry D. (2025). Degradation of Polyester Coil-Coated Materials by Accelerated Weathering Investigated by FTIR-ATR Chemical Imaging and Impedance Analysis. Prog. Org. Coat..

[28] Makki H., Adema K.N.S., Peters E.A.J.F., Laven J., Van Der Ven L.G.J., Van Benthem R.A.T.M., De With G. (2015). Quantitative Spectroscopic Analysis of Weathering of Polyester-Urethane Coatings. Polym. Degrad. Stab..

[29] Nandiyanto A.B.D., Ragadhita R., Fiandini M. (2022). Interpretation of Fourier Transform Infrared Spectra (FTIR): A Practical Approach in the Polymer/Plastic Thermal Decomposition. Indones. J. Sci. Technol..

[30] Sawada R., Liu H., Ando S. (2025). Vibrational Spectroscopic Analysis of Water Absorption in Polyimides and the Correlation with Dielectric Properties at 10 GHz. J. Phys. Chem. B.

[31] Campanale C., Savino I., Massarelli C., Uricchio V.F. (2023). Fourier Transform Infrared Spectroscopy to Assess the Degree of Alteration of Artificially Aged and Environmentally Weathered Microplastics. Polymers.

[32] La Russa M.F., Ruffolo S.A., Barone G., Crisci G.M., Mazzoleni P., Pezzino A. (2009). The Use of FTIR and Micro-FTIR Spectroscopy: An Example of Application to Cultural Heritage. Int. J. Spectrosc..

[33] Rampazzi L., Andreotti A., Bonaduce I., Colombini M.P., Colombo C., Toniolo L. (2004). Analytical Investigation of Calcium Oxalate Films on Marble Monuments. Talanta.

[34] Ginsberg S.A., Rayburn L.P., Bray A.J., Nuñuz-Parker F.S., Dowling A., Russ J. (2023). Trace Organic Compounds in Oxalate Rock Accretions from the Lower Pecos Canyonlands of Southwestern Texas. Geoarchaeology.

[35] Scheerer S., Ortega-Morales O., Gaylarde C. (2009). Microbial Deterioration of Stone Monuments—An Updated Overview. Adv. App. Microbiol..

[36] Liu X., Koestler R.J., Warscheid T., Katayama Y., Gu J.-D. (2020). Microbial Deterioration and Sustainable Conservation of Stone Monuments and Buildings. Nat. Sustain..

[37] Singh M.R., Yadav R. (2023). Formation of Calcium Oxalate Patinas as Protective Layer on Basaltic Stone Surfaces of 17th Century Raigad Hill Fort, India. Heritage.

[38] Chang C., Feng L.-F., Gu X.-P., Zhang C.-L., Dai L.-K., Chen X., Hu G.-H. (2022). In Situ Raman Spectroscopy Real-Time Monitoring of a Polyester Polymerization Process for Subsequent Process Optimization and Control. Ind. Eng. Chem. Res..

[39] Conti C., Aliatis I., Colombo C., Greco M., Possenti E., Realini M., Castiglioni C., Zerbi G. (2012). μ-Raman Mapping to Study Calcium Oxalate Historical Films. J. Raman Spectrosc..

[40] Pinlova B., Nowack B. (2024). From Cracks to Secondary Microplastics—Surface Characterization of Polyethylene Terephthalate (PET) during Weathering. Chemosphere.

[41] Ainali N.M., Bikiaris D.N., Lambropoulou D.A. (2024). Physicochemical Alterations on UV Aged Polymers Leading to Microplastics Formation: A Multi-Tiered Study of Polyester, Polycarbonate and Polyamide. Polym. Degrad. Stab..

[42] Meng J., Wang Y., Yang H., Wang P., Lei Q., Shi H., Lei H., Fang D. (2020). Mechanical Properties and Internal Microdefects Evolution of Carbon Fiber Reinforced Polymer Composites: Cryogenic Temperature and Thermocycling Effects. Compos. Sci. Technol..

[43] Liao J., Yang L., Chen Z., Guan T., Liu T. (2023). Effect of Thermal Cycling on Microstructure and Mechanical Properties of C_f_ /SiC–Al Composites. Adv. Eng. Mater..

[44] Yılmaz Atalı P., Doğu Kaya B., Manav Özen A., Tarçın B., Şenol A.A., Tüter Bayraktar E., Korkut B., Bilgin Göçmen G., Tağtekin D., Türkmen C. (2022). Assessment of Micro-Hardness, Degree of Conversion, and Flexural Strength for Single-Shade Universal Resin Composites. Polymers.

[45] Xu B., Van Den Hurk B., Liu T., Blok R., Teuffel P. (2024). Effect of UV-Water Weathering on the Mechanical Properties of Flax-Fiber-Reinforced Polymer Composites. Polym. Compos..

[46] González-Alenda E., Baracco B., Perdigão J., Jiménez-Díez D., Garrido M.Á., Álvarez-Lloret P., Ceballos L., Fuentes V. (2025). Physicomechanical Properties and Morphological Characterization of Several Universal Resin Composites After Different Aging Procedures. J. Esthet. Restor. Dent..

[47] Hatami Naderloo S., Mohebby B., Kazemi Najafi S. (2024). Effect of Natural Weathering on Performance of Wood Flour-Recycled Polypropylene Composites. Drv. Ind..

[48] Bakshi P., Pappu A., Bharti D.K., Patidar R. (2021). Accelerated Weathering Performance of Injection Moulded PP and LDPE Composites Reinforced with Calcium Rich Waste Resources. Polym. Degrad. Stab..

[49] Singer G., Sinn G., Schwendtner K., Lichtenegger H.C., Wan-Wendner R. (2018). Time-Dependent Changes of Mechanical Properties of Polymer-Based Composite Materials for Adhesive Anchor Systems. Compos. Struct..

[50] Golewski P., Sadowski T., Kneć M., Budka M. (2023). The Effect of Thermal Aging Degradation of CFRP Composite on Its Mechanical Properties Using Destructive and Non-Destructive Methods and the DIC System. Polym. Test..

[51] Huang Z., Chen B., Ren B., Tu D., Wang Z., Wang C., Zheng Y., Li X., Wang D., Ren Z. (2023). Smart Mechanoluminescent Phosphors: A Review of Strontium-Aluminate-Based Materials, Properties, and Their Advanced Application Technologies. Adv. Sci..

[52] Aktug Karademir S., Atasoy S., Akarsu S., Karaaslan E. (2025). Effects of Post-Curing Conditions on Degree of Conversion, Microhardness, and Stainability of 3D Printed Permanent Resins. BMC Oral Health.

[53] Mohammadi H., Morovati V., Korayem A.-E., Poshtan E., Dargazany R. (2021). Constitutive Modeling of Elastomers during Photo- and Thermo-Oxidative Aging. Polym. Degrad. Stab..

[54] Mahmudzade R., Nikaeen P., Chirdon W., Khattab A., Depan D. (2022). Photodegradation Mechanisms and Physico-Chemical Properties of EPON-IPD Epoxy-Based Polymers. React. Funct. Polym..

[55] Kumar D.S., Shukla M.J., Mahato K.K., Rathore D.K., Prusty R.K., Ray B.C. (2015). Effect of Post-Curing on Thermal and Mechanical Behavior of GFRP Composites. IOP Conf. Ser. Mater. Sci. Eng..

[56] Jahani Y., Baena M., Barris C., Perera R., Torres L. (2022). Influence of Curing, Post-Curing and Testing Temperatures on Mechanical Properties of a Structural Adhesive. Constr. Build. Mater..

[57] Naser Alavi F., Ghavami-Lahiji M., Habibi P. (2023). Mechanical Performance of a Conventional Resin Composite and Its Bulk-Fill Restorative Counterpart after Long-Term Accelerated Aging. Dent. Med. Probl..

[58] Kim S., Lee Y., Kim C., Choi S. (2022). Analysis of Mechanical Property Degradation of Outdoor Weather-Exposed Polymers. Polymers.

[59] Yeşilay Kaya S., Karacaoglu E., Karasu B. (2012). Effect of Al/Sr Ratio on the Luminescence Properties of SrAl2O4:Eu2+, Dy3+ Phosphors. Ceram. Int..

[60] Suda Y., Okuno T., Takeda T., Takahashi K., Hirosaki N. (2024). The Decay Curves of Luminescence from Eu^2+^ in *β* -SiAlON Are Effectively Analyzed Using the General-Order Kinetics Formula. J. Phys. D Appl. Phys..

[61] Zhu Y., Zheng M., Zeng J., Xiao Y., Liu Y. (2009). Luminescence Enhancing Encapsulation for Strontium Aluminate Phosphors with Phosphate. Mater. Chem. Phys..

[62] Tsai C.-Y., Lin J.-W., Huang Y.-P., Huang Y.-C. (2015). Experimental Modeling and Evaluation of the Afterglow Phosphors Using Multiple Single Exponential Equations. Neurocomputing.

[63] Guo X., Zhang K., Ge M. (2019). The Luminous Mechanism of Eu^2+^ and Dy^3+^ Co-Doped Long Persistent Luminous Fiber. Text. Res. J..

[64] Sawan S.E.A., Hamdy Y.M., Khattab R.M. (2025). Investigation of the Phosphorescence, Persistent Decay and Structure Properties of Eu2+: Strontium Aluminate Doped with Nd3+, B3+ or Dy3+. BMC Chem..

[65] Swart H.C., Terblans J.J., Ntwaeaborwa O.M., Kroon R.E., Mothudi B.M. (2012). PL and CL Degradation and Characteristics of SrAl2O4: Eu2+,Dy3+ Phosphors. Phys. B Condens. Matter.

[66] Khattab T.A., Abd El-Aziz M., Abdelrahman M.S., El-Zawahry M., Kamel S. (2020). Development of Long-persistent Photoluminescent Epoxy Resin Immobilized with Europium (II)-doped Strontium Aluminate. Luminescence.

[67] Li X., Jin B.C., Tsotsis T.K., Nutt S. (2022). Thermo-Oxidative Aging and Thermal Cycling of PETI-340M Composites. High Perform. Polym..

[68] Havasi V., Tátrai D., Szabó G., Varga E., Erdőhelyi A., Sipos G., Kónya Z., Kukovecz Á. (2020). On the Effects of Milling and Thermal Regeneration on the Luminescence Properties of Eu2+ and Dy3+ Doped Strontium Aluminate Phosphors. J. Lumin..

[69] Essahili O., Ouafi M., Ilsouk M., Lakbita O., Duhayon C., Mahi L., Moudam O. (2024). Photoluminescence Lifetime Stability Studies of B-diketonate Europium Complexes Based Phenanthroline Derivatives in Poly(Methyl Methacrylate) Films. ChemistryOpen.

[70] Al-Qahtani S.D., Alshareef M., Aljohani M., Alhasani M., Felaly R., Habeebullah T.M., El-Metwaly N.M. (2022). Simple Preparation of Photoluminescent and Color-Tunable Polyester Resin Blended with Alkaline-Earth-Activated Aluminate Nanoparticles. ACS Omega.

[71] Abdu M.T., Khattab T.A., Abdelrahman M.S. (2023). Development of Photoluminescent and Photochromic Polyester Nanocomposite Reinforced with Electrospun Glass Nanofibers. Polymers.

[72] Poulose A.M., Anis A., Shaikh H., Alhamidi A., Siva Kumar N., Elnour A.Y., Al-Zahrani S.M. (2021). Strontium Aluminate-Based Long Afterglow PP Composites: Phosphorescence, Thermal, and Mechanical Characteristics. Polymers.

[73] Sandt C., Waeytens J., Deniset-Besseau A., Nielsen-Leroux C., Réjasse A. (2021). Use and Misuse of FTIR Spectroscopy for Studying the Bio-Oxidation of Plastics. Spectrochim. Acta Part A Mol. Biomol. Spectrosc..

[74] Persson D., Heydari G., Edvinsson C., Sundell P.E. (2020). Depth-Resolved FTIR Focal Plane Array (FPA) Spectroscopic Imaging of the Loss of Melamine Functionality of Polyester Melamine Coating after Accelerated and Natural Weathering. Polym. Test..

[75] Krieg T., Mazzon C., Gómez-Sánchez E. (2021). Material Analysis and a Visual Guide of Degradation Phenomena in Historical Synthetic Polymers as Tools to Follow Ageing Processes in Industrial Heritage Collections. Polymers.

[76] Jaszczur M., Teneta J., Styszko K., Hassan Q., Burzyńska P., Marcinek E., Łopian N. (2019). The Field Experiments and Model of the Natural Dust Deposition Effects on Photovoltaic Module Efficiency. Environ. Sci. Pollut. Res..

[77] Younis A., Cotfas P.A., Cotfas D.T. (2024). Systematic Indoor Experimental Practices for Simulating and Investigating Dust Deposition Effects on Photovoltaic Surfaces: A Review. Energy Strategy Rev..

[78] Hassan G., Sami Yilbas B., Al-Sharafi A., Al-Sulaiman F., Abdulhamid Abubakar A. (2024). Dust Mitigation Strategies Concerning Solar Energy Applications: A Comprehensive Review. Sol. Energy.

[79] Kim H.-K., Kim S.-H., Lee J.-B., Han J.-S., Yeo I.-S. (2013). Effect of Polishing and Glazing on the Color and Spectral Distribution of Monolithic Zirconia. J. Adv. Prosthodont.

[80] Brescansin F.N., Prochnow C., Guilardi L.F., Kleverlaan C.J., Bacchi A., Valandro L.F., Pereira G.K.R. (2021). Effect of Different Surface Treatments on Optical, Colorimetric, and Surface Characteristics of a Lithium Disilicate Glass–Ceramic. J. Esthet. Restor. Dent..

[81] Reiß L., Prestel T., Giering S. (2022). The Light Aging Behavior of Daylight Fluorescent Paints: A Colorimetric, Photographic, Raman Spectroscopic and Fluorescence Spectroscopic Study. Herit. Sci..

[82] Huang J., Fu P., Li W., Xiao L., Chen J., Nie X. (2022). Influence of Crosslinking Density on the Mechanical and Thermal Properties of Plant Oil-Based Epoxy Resin. RSC Adv..

