# Supplementary material for: Structural and Functional Stability of Strontium Aluminate-Based Luminescent Composites After 15 Years of Natural Weathering (Indoors and Outdoors)
Source: Polymers (Basel). 2026 Aug 9;18(16):1949. doi: 10.3390/polym18161949 (PMC13516463; doi:10.3390/polym18161949)
Supplement: Supplementary file 1 [file polymers-18-01949-s001.zip › polymers-4431848-supplementary.pdf]

Supplementary Materials

Mª Ángeles Rodríguez-González [1,*], Natalia Díaz-Rodríguez [2], Miguel Rubio-Carrizo [3] and Fausto Rubio [3]

[1] Environmental Resources Analysis Research Group, University of Extremadura, 06006 Badajoz, Spain
[2] Biochemistry and Molecular Biology and Genetics Department, University of Extremadura, 06006 Badajoz, Spain; nataliadr@unex.es
[3] Chemistry-Physics of Surfaces and Processes Department, Institute of Ceramics and Glass (CSIC), 28049 Madrid, Spain; miguel.rubio@icv.csic.es (M.R.-C.); frubio@icv.csic.es (F.R.)
* Correspondence: marodgon@unex.es

Table of Content

Figure S1. Elemental EDS Mapping of the 15-year outdoor weathered sample before polishing.

Figure S2. Elemental EDS Mapping of the 15-year outdoor weathered sample after polishing.

Figure S3. Profilometric surface profiles of the 15-year outdoor weathered sample before polishing.

Figure S4. Profilometric surface profiles of the 15-year outdoor weathered sample after polishing.

Table S1. Roughness parameters of the 15-year outdoor weathered sample before polishing.

Table S2. Roughness parameters of the 15-year outdoor weathered sample after polishing.

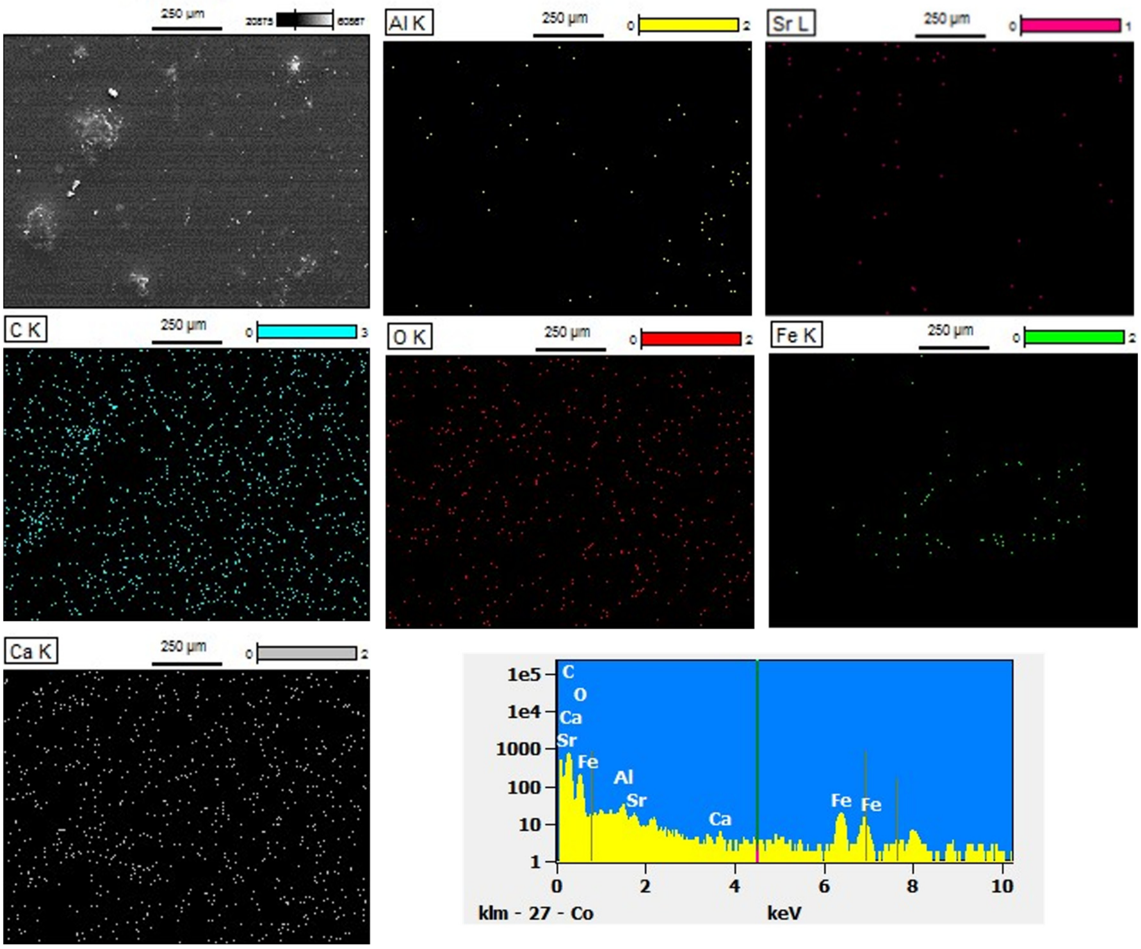

Figure S1. Elemental EDS Mapping of the 15-year outdoor weathered sample before polishing.

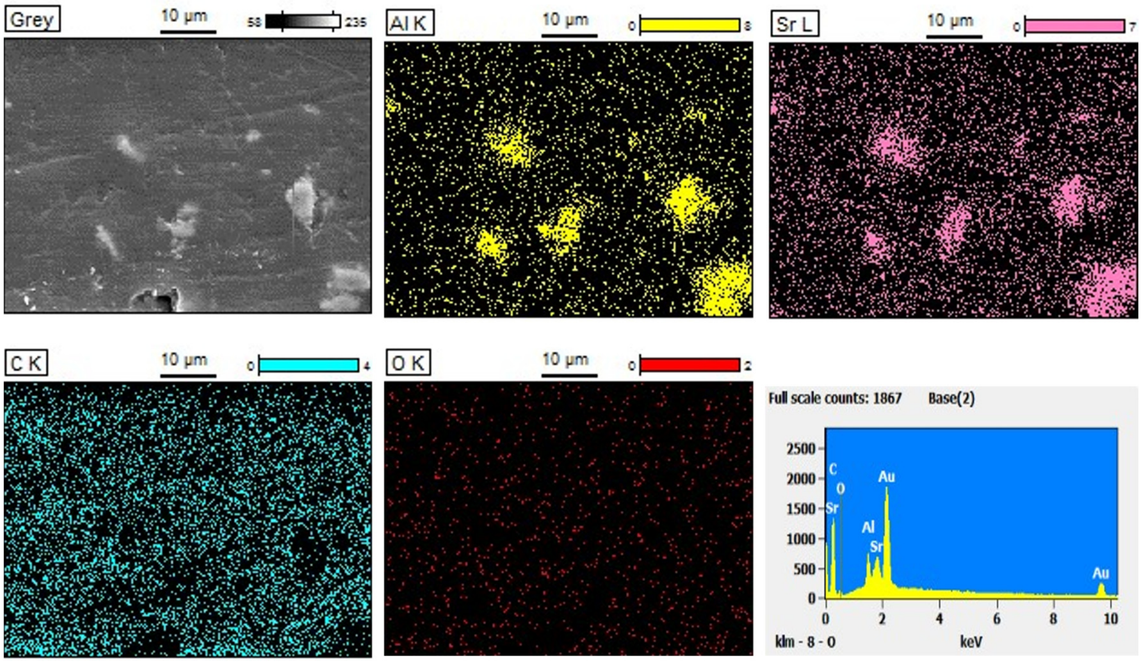

Figure S2. Elemental EDS Mapping of the 15-year outdoor weathered sample after polishing.

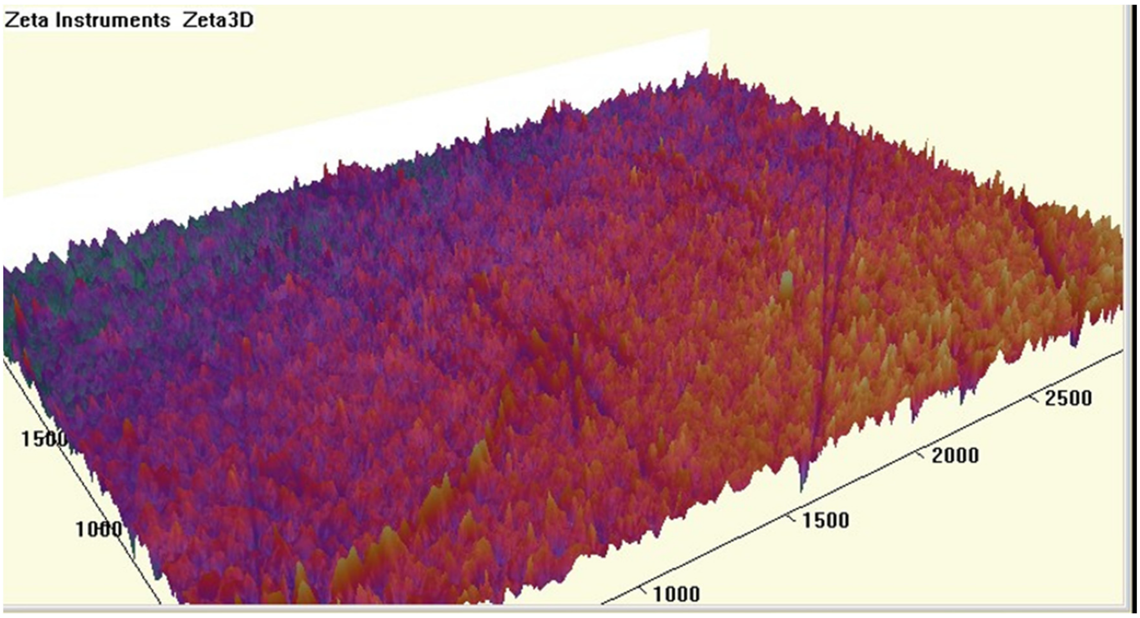

Figure S3. Profilometric surface profiles of the 15-year outdoor weathered sample before polishing.

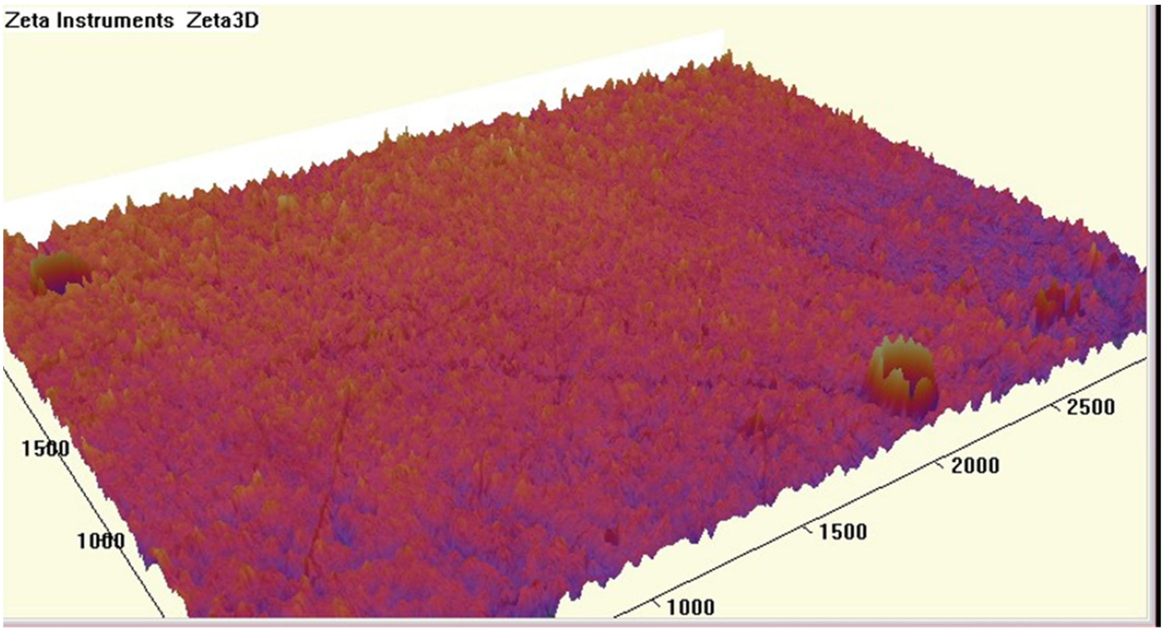

Figure S4. Profilometric surface profiles of the 15-year outdoor weathered sample after polishing.

Table S1. Roughness parameters of the 15-year outdoor weathered sample before polishing.

	Unpolished (µm)							
Section	Ra	Rq	Rpv	Rp	Rv	Rsk	Rz	Rku
1	11.35	14.20	89.13	52.35	36.78	0.0422	73.47	0.248
2	9.75	12.24	73.32	32.38	40.94	$0.865.10^{-3}$	60.96	0.244
3	9.43	12.19	83.60	43.34	40.26	0.0169	68.64	0.394
4	10.46	12.98	84.93	30.36	54.57	-0.057	64.61	0.364
5	10.23	13.29	99.04	53.10	45.94	0.0163	80.88	0.367
6	11.09	14.25	97.56	40.15	57.40	-0.0415	79.81	0.329
7	9.80	12.31	73.38	32.98	40.40	-0.0078	63.77	0,255
8	10.16	13.00	85.40	49.33	36.07	-0.0032	68.68	0.281
9	9.31	12.73	93.14	34.82	58.32	-0.0777	73.52	0.496
10	8.56	10.79	78.35	34.41	43.94	-0.0221	58.92	0.361
11	12.27	16.19	100.24	34.82	65.42	-0.09	78.10	0.441
AVERAGE	10.22	13.11	87.10	39.82	47.28	-0.02	70.12	0.34

Table S2. Roughness parameters of the 15-year outdoor weathered sample after polishing.

	Polished (µm)							
Section	Ra	Rq	Rpv	Rp	Rv	Rsk	Rz	Rku
1	9.71	12.12	75.93	35.83	40.10	-0.0168	59.58	0.299
2	8.84	11.16	70.38	30.80	39.59	-0.0247	57.12	0.312
3	8.27	10.16	55.47	29.06	26.42	0.0157	52.66	0.260
4	8.31	10.66	72.81	42.58	30.23	0.0370	59.83	0.307
5	7.95	10.31	84.22	41.93	42.29	0.0263	57.55	0.323
6	7.80	10.38	103.01	60.50	42.50	0.0319	61.69	0.637
7	7.39	9.59	81.55	29.83	51.73	-0.0092	55.28	0,500
8	7.74	10.13	93.43	39.43	54.01	-0.0621	60.91	0.613
9	9.83	12.43	81.72	44.68	37.04	-0.0104	65.53	0.411
AVERAGE	8.43	10.77	79.84	39.40	40.43	0.00	58.91	0.41
